# Evaluation and Potential Uses of Site Investigation Soil Contamination Data Submitted to Uk Local Government

**DOI:** 10.1007/s00267-022-01648-1

**Published:** 2022-05-04

**Authors:** Emma E. Hellawell, Susan J. Hughes

**Affiliations:** 1 Department of Civil and Environmental Engineering, University of Surrey, Guildford, GU2 7XH UK; 2 University of Surrey, Guildford, GU2 7XH UK

**Keywords:** Contaminated land, Site investigation, Brownfield site, Local government

## Abstract

There has been considerable brownfield development in the UK since 2000 due to increasing demand for new housing, combined with local opposition to building on greenbelt land. To facilitate this, extensive site investigations have been carried out and the reports submitted to local government as part of the planning process. This research investigates whether this largely untapped resource of site investigation data can be used to improve understanding of potentially toxic elements (PTE) and persistent organic pollutants (POP) at a local scale. The PTE/POP data were extracted from 1707 soil samples across 120 brownfield sites in an urban/suburban region. The samples were analysed to determine the effect of site location, historical use and site age on PTE/POP concentrations. Box plots indicating statistical results together with GIS maps of PTE/POP sample data provided the optimal visualisation of results. The dataset was shown to be a valuable resource, although further exploitation would be enhanced by digitisation of the submitted data. The paper explores potential applications of this data, including background concentrations and anthropogenic enrichment factors for PTE/POP. The results were summarised in a table for the PTE/POP and a preliminary risk assessment process chart to inform developers/regulators on potential PTE/POP levels on brownfield sites on a local scale. This information could focus design and resources for developers for site investigations and risk assessments and improve planning and regulatory guidance. The lack of predictability in PTE/POP results across sites have emphasised the ongoing need for intrusive site investigation on new brownfield developments.

## Introduction

In 1998, the UK government set a national target for 60 percent of all new developments to be on brownfield land (Environment, Transport and Regional Affairs Committee 1998). This political aim was incorporated into planning policy and consequently, in the last 20 years, considerable development has taken place on brownfield land. To facilitate this, extensive site investigations have been carried out and the reports on these are submitted to local government as part of the planning process. Following completion of the development, this expensive and potentially valuable resource of environmental data is stored in local government databases throughout England and Wales. This paper provides an analysis of these data for a local government area and discusses how this information can be used to improve knowledge of soil contamination and apprise brownfield development policy.

The UK local government dataset was first interrogated in Gateshead, northeast England (Rothwell and Cooke 2015) to evaluate background levels of metal contamination and has recently been evaluated in Surrey for asbestos and polyaromatic hydrocarbons (PAH) contamination (Hellawell and Hughes 2021). These studies together with a further data assessment study (Hellawell and Hughes 2020) found that these data were a useful resource to evaluate local levels of potentially toxic elements (PTE) and persistent organic pollutants (POP) such as PAH. The site data in these studies dated back to 1996, however there were issues raised relating to the reliability of older data as laboratory analytical methods have developed and improved over time.

Concentrations of potentially toxic elements (PTE) on a UK national scale for key contaminants have been investigated by an extensive British Geological Survey study (Ander et al. 2013). This work was extended to several urban areas, with a sampling strategy of four samples per square kilometre (Ferreira et al. 2017; Lark and Scheib 2013; McIlwaine et al. 2017). A similar sampling strategy was also used for PAH and polychlorinated biphenyls (PCB) contamination in surface soils in an area of Greater London (Vane et al. 2014). There is, however, currently a lack of useful available information on soil contamination at a local scale in the UK, with this potential government resource largely unexplored.

Internationally, there have been regional and national studies of PTE levels (for example in Greece, Massas et al. 2013; China, Yang et al. 2009; Australia, Reimann, de Caritat (2017)) These particular studies measured PTE concentrations in shallow soils and compared these to a representative value for a subsurface sample, thus evaluating an enrichment factor relating anthropogenic to geogenic concentrations of PTE. Reimann, de Caritat (2017) state that this enrichment factor or ratio may only be relevant for brownfield sites or mining areas. Regional PTE information from previous studies has been statistically analysed and used to evaluate background and threshold concentrations of PTEs (Ander et al. 2013; Jarva et al. 2010; Reimann et al. 2005; Rothwell and Cooke 2015). These background and threshold concepts which originate from environmental geochemistry are intended to differentiate between natural geological background and anthropogenic influences. These are then used to indicate if and when further investigation or remediation is required. There are, however, differences in the definitions and evaluation methods for calculating the background and threshold values, which vary with application, scale and national remits (Reimann, de Caritat 2017). In the UK, the requirement for determining local ‘normal’ background levels was introduced into UK legislation by Defra Guidance in 2012 (DEFRA 2012). This part of the 2012 legislation has not been practically implemented in local government due to a lack of clear method and limited local government resources to collect and analyse locally measured soil data.

This research project investigates how the largely untapped resource of site investigation data that is already available from planning applications can be used to improve understanding of PTE/POP concentrations at a local scale. These data could become a valuable resource for local government and developers to inform policy and development on brownfield sites in the UK and worldwide. To achieve this the following objectives were identified and are presented in this paper.

i. Extrapolation and statistical evaluation of data on brownfield sites taken from local government planning applications to understand the scope and scale of PTE/POP contamination.
ii. Data analysis to determine the effect of site location, historical site use and site age on PTE/POP concentrations
iii. Explore potential applications and uses of these data, including background concentrations and anthropological enrichment factors for PTE/POP, to inform local government.

## Background

Two hundred years of industrial development and urbanisation in the UK has left a legacy of contaminated land. Sites that have previously been used for industrial, commercial or residential purposes are categorised as brownfield (Fraser et al. 2018). The activities on these sites and general waste disposal have left a layer of Made Ground, consisting of fill, infill, site debris or topsoil often containing material such as ash, coal waste, bricks, etc. UK legislation defining the legal framework for dealing with contaminated land was introduced in the 1990s. The UK Statutory Guidance for enacting this framework is outlined in DEFRA 2012. The main objectives of this legislation are to identify and reduce ‘unacceptable risk to humans and the environment’ and ensure that sites are ‘suitable for use’. These concepts were included in planning legislation and hence the contamination risks on any development site are evaluated as part of the local government planning process (NPPF 2019). The local government planning officer and contaminated land officer, set and review these planning requirements through planning conditions that require compliance for planning approval.

The first requirement in the planning process is a desk study of the site detailing the history, geology and industrial activity of the site and surrounding area. This desk study and site walkover visit then informs a conceptual site model (CSM) which evaluates the potential risk for the site and its proposed use. A risk is identified if there is one or more pollutant–receptor linkage. An intrusive site investigation is then required to update the site CSM and to provide the required information for a qualitative (and if needed, quantitative) risk assessment. Further stages in this planning process include a remediation method statement, detailing the procedure that will be followed to mitigate the risk and a verification report (Environment Agency 2004, 2019). The final verification report (carried out by an independent consultant) confirms that the approved remediation has been carried out and may include further soil/water/gas sampling to verify the site is suitable for its use. All these reports are submitted to local government for approval to enable the planning conditions to be discharged.

Through this planning process each local planning authority in the UK has details on almost all of the site investigations undertaken in their area, since 2000. This information is submitted to local government and is available for public viewing at the council, on planning websites or can be requested under the Environmental Information Regulations (2004). The data are, however, submitted in hard copy or pdf format, in large reports with extensive appendices. Access to these data is time consuming as the planning database is not categorised to enable easy extraction of these site reports. Within councils, potentially contaminated sites are detailed through a GIS database, with links to these planning reports; however this database is not available to the public. The site investigation reports are not digitised and the relevant data requires manual extraction. Hence, there is no current system for obtaining and extracting this key information from reports (within or external to local government) for regional contamination analysis. Whilst the site reports can be requested through Environmental Information Regulations (2004), this process may incur an administrative charge.

## Method

To benchmark the viability of using these data (outside its original remit of securing local planning permissions) a case study region with sufficient site data was required. An ideal area would be where demand for residential development is high and there is a lack of greenfield options (i.e. due to protected greenbelt), hence there has been significant brownfield development. It was also considered important that the area was not tied to a particular past industrial use and was therefore representative of a UK suburban area. As part of the re-development, any brownfield land would have undergone an intrusive site investigation to secure the required planning permission and there would be archived site data available for the analysis in this study. The region governed by Elmbridge Borough Council met these requirements.

Elmbridge and its environs encompass a suburban area of ~9600 ha, lying 20 km southwest of London, with a population of ~131,000 (Elmbridge Borough Council 2015). It is an affluent commuter area for London, where urban expansion is protected by green belt and any available (and appropriate) brownfield land is considered for residential development. The area is not historically tied to a former industry: previous site uses include gravel extraction, aviation works, motor sports, water treatment, waste infill and numerous small engineering and industrial works.

Once selected, the local authority database of potentially contaminated sites, (as defined under UK Part 2A Environmental Protection Act 1990 legislation) was interrogated, to find the development sites with intrusive site investigation reports. In Elmbridge, this database provided links or planning numbers that could then be used to find the required site information and associated reports on the planning website (Elmbridge Borough Council 2018). Finding and extracting the reports was time consuming. A few reports, generally from site investigations prior to 2012, had not been uploaded on the website, but were provided by the council. The data, even for the latest reports, were not in an accessible digital format, but instead in the form of scanned hardcopies of reports, chemical spreadsheets and maps. Table 1 details the information and sites used for this study.

**Table 1:** Information available

Information	Number
Sites with intrusive site investigation data	120
Range of dates of investigations	1996–2019
Number of boreholes/ window samples etc.	1188
Number of soil samples tested	1707

Data from all the relevant reports were manually entered into a relational database. This was constructed based upon Ander et al. (2013) and Rothwell and Cooke (2015) and included information on site history/past use, site geology, current land use, results of chemical analysis on the soil samples and the laboratory, chemical tests used and data quality (Hellawell and Hughes 2020).

The soil samples were tested for a range of PTE/POP, based upon the conceptual site model (CSM), the consultant’s requirement and the budget. The standard core suite of chemicals tested included arsenic, boron, cadmium, chromium (total), copper, lead, mercury, nickel, selenium, zinc, 16 PAHs and total petroleum hydrocarbons (TPH). Selection of testing for other chemicals was ad hoc and the data, therefore, contained a lower number of measurements for cyanide, sulphate, total organic carbon, total organic matter, phenols, chromium hexavalent and vanadium. Due to the use of different laboratories and test methods for chemical analysis, there were some recognised inconsistencies in the dataset. Information was provided in the reports on the test procedures and whether an approved laboratory/method (e.g. MCERTS in the UK for Environment Agency approved testing in line with EN ISO/IEC 17025) was used. Any discrepancies were noted in the dataset.

The location of sampling points within the Case Study region depended upon the locations of development sites. Over 70% of sites were developed for residential use and 95% of sites were within urban or sub-urban areas (Supplementary Information, S1 details this).

Information on the site history and previous site use were entered into the database for each site. This information was generally obtained from desk study reports, also submitted to local government during the planning process. Where this information was not available, historic Ordnance Survey maps, dating back to 1870 were interrogated though the EDINA Historic Digimap Service (2019). The date the site was developed was recorded. This was taken as the time at which significant development at the site appeared on the Ordnance Survey maps. Prior uses, for example, where the site was part of a manor house were not included; instead the date taken was when workshops or industrial units were first indicated.

The database was then analysed for the following:

- Statistical evaluation of PTE/POP for Made Ground and natural soil strata
- The location of PTE/POP found within the study region
- The impact of site historic use and the age the site was developed on measured PTE/POP concentrations in Made Ground.

## Results

### Evaluation of PTE/POP Concentrations and Location in Elmbridge

This section outlines the general results of the dataset from Elmbridge Borough Council for the main PTE analysed in the Case Study region. These are presented in terms of the mean and median concentrations for the analytes and their distribution within the local authority area for all the Made Ground and natural soil samples.

As shown in Table 2, each site had differing numbers of samples; hence the results could be skewed by sites with a large number of samples. To assess the extent of this, a second overall site mean and median were calculated from the mean and median of each site. Both sets of results (see Supplementary Information S2) showed similar trends which confirmed any skewing of data associated with an uneven sampling distribution was minimal. Reimann et al. (2005) noted that the mean values were more likely to be affected by a few outlier data points and hence for contaminated land analysis, the median results were generally considered more appropriate. This was also the case for this dataset in which the mean results were all greater than the values for the medians, due to the outliers.

**Table 2:** Results for PTE and POP concentrations measured in Made Ground and natural soil samples

Chemical	Made Ground					Natural soil				
	Number of samples	% at limit of detection	Number exceeding guidance^a^	Mean	Median	Number of samples	% at limit of detection	Number exceeding guidance^a^	Mean	Median
*Units mg/kg unless stated*										
Antimony	46	24		2.8	2.0	16	56		1.3	*1.0*
Arsenic	1008	2	37	16.0	13.0	481	2	13	13.5	11.0
Beryllium	238	35	15	1.1	1.0	98	50	12	1.4	*1.0*
Water soluble Boron	715	37	0	1.7	1.0	355	53	0	1.1	*0.8*
Cadmium	1008	53	0	0.9	*0.5*	479	76	0	0.6	*0.5*
Chromium	1008	0	0	28.2	22.0	479	0	0	28.1	23.0
Chromium (hexavalent)	351	98	0	1.9	*1.0*	116	98	0	2.0	*2.0*
Copper	1005	3	8	137.3	27.0	479	8	1	30.5	9.2
Lead	1008	0	321	**255.0**	110.0	481	4	27	97.8	18.0
Mercury	1008	61	62	0.7	*0.5*	479	81	0	0.5	*0.3*
Nickel	1008	2	3	21.8	17.0	479	4	1	18.8	15.0
Selenium	984	82	0	1.4	*1.0*	465	82	0	1.3	*1.0*
Vanadium	279	0	0	38.4	36.0	116	0	0	39.8	37.4
Zinc	1000	0	3	200.5	94.0	477	1	0	82.3	41.0
Total Cyanide	808	82		36.7	*1.0*	352	93		2.2	*1.0*
Sulphate as SO4 (g/l)	587	14		9.6	0.1	232	20		2.0	0.0
Sulphide	430	67		120.4	*10.0*	239	89		25.2	*10.0*
Organic Matter %	245	3		3.1	2.3	91	9		1.8	1.4
Total Organic Carbon %	365	4		2.0	1.2	124	23		0.8	0.4
Total Phenols (monohydric)	807	86	0	1.4	*1.0*	357	96	0	0.9	*1.0*
Total Petroleum Hydrocarbons (TPH)	349	41		376.9	45.0	208	63		244.5	31.9
Benzo(a)pyrene (BaP)	755	26	112	**8.8**	0.5	346	68	6	1.7	0.1
Total PAH	827	27		114.3	6.8	379	68		115.7	1.6

Bold values show where a value exceeds the current UK guidance for residential use with plant uptake. Italic indicates where median is also the limit of detection for the PTE

^a^Guidance is either C4SL or S4UL for residential use with plant uptake (DEFRA 2014; Nathanail et al. 2015)

The dataset contained many samples that were determined by the laboratory to be below the limit of detection (LOD) of the analytical method. These samples were important in showing where low concentrations of PTE/POP were found, however they could also distort statistical results. To include these data, the worst-case scenario of the limit of detection was allocated. A parametric study using a range of LOD values was carried out. The only significant effect on results was noted when the median result was the allocated limit of detection for the PTE/POP. These values are highlighted using italics in Table 2.

The key results from Table 2 relating to the sample set are those for copper, lead, zinc, cyanide, sulphide, total petroleum hydrocarbons (TPH), benzo(a)pyrene (BaP) and total polycyclic aromatic hydrocarbons (PAH):

- the concentrations of these PTE/POPs measured in natural soil were significantly lower than those for Made Ground, indicating the anthropogenic source of the contamination in this region.

- the mean values of these PTE/POPs were significantly higher than the medians. This indicated that there were a number of high-concentration outliers for these PTE/POP, which skewed the mean results.

**Fig. 1:**
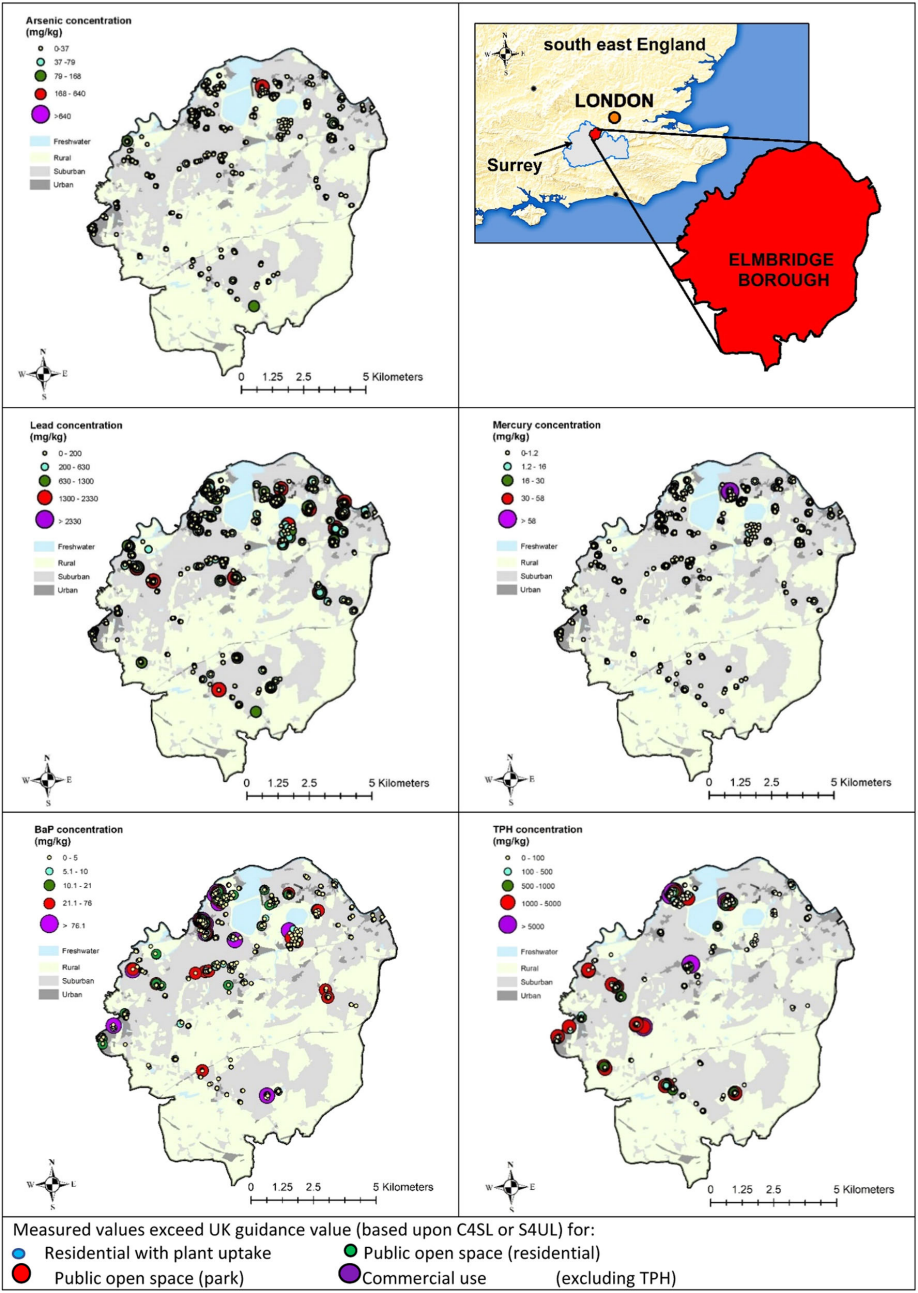
Plots for PTE on brownfield sites with exceedances of UK guidance levels. (Service Layer Credits: Contains UK data ©Crown Copyright and Database Right 2021 and Land Cover Map from Rowland et al. 2017)

The spatial distribution of the key PTE/POP, with exceedances of guidance levels (C4SL or appropriate S4UL levels: DEFRA 2014, Nathanail et al. 2015) for all sampling points was overlaid upon a map showing urban, suburban and rural land uses using ArcVIEW GIS. Elmbridge Borough lies southwest of London and becomes more developed in the northeast of the Borough. Figure 1 shows that most of the sites were in urban and suburban areas of the Borough. These maps provide a visual interpretation of the data that is summarised in Table 2. The contaminant concentrations detailed in the legends were selected based upon current UK guidance (C4SL or appropriate S4UL levels: DEFRA 2014, Nathanail et al. 2015). There were no obvious correlations between the different PTE/POP on these sites. Samples with elevated lead concentrations did not show corresponding high levels of TPH, indicating petroleum was probably not the source of elevated lead in this area.

### Effect of Former Site Use and Site Age on PTE/Organic Pollutant in Elmbridge

**Fig. 2:**
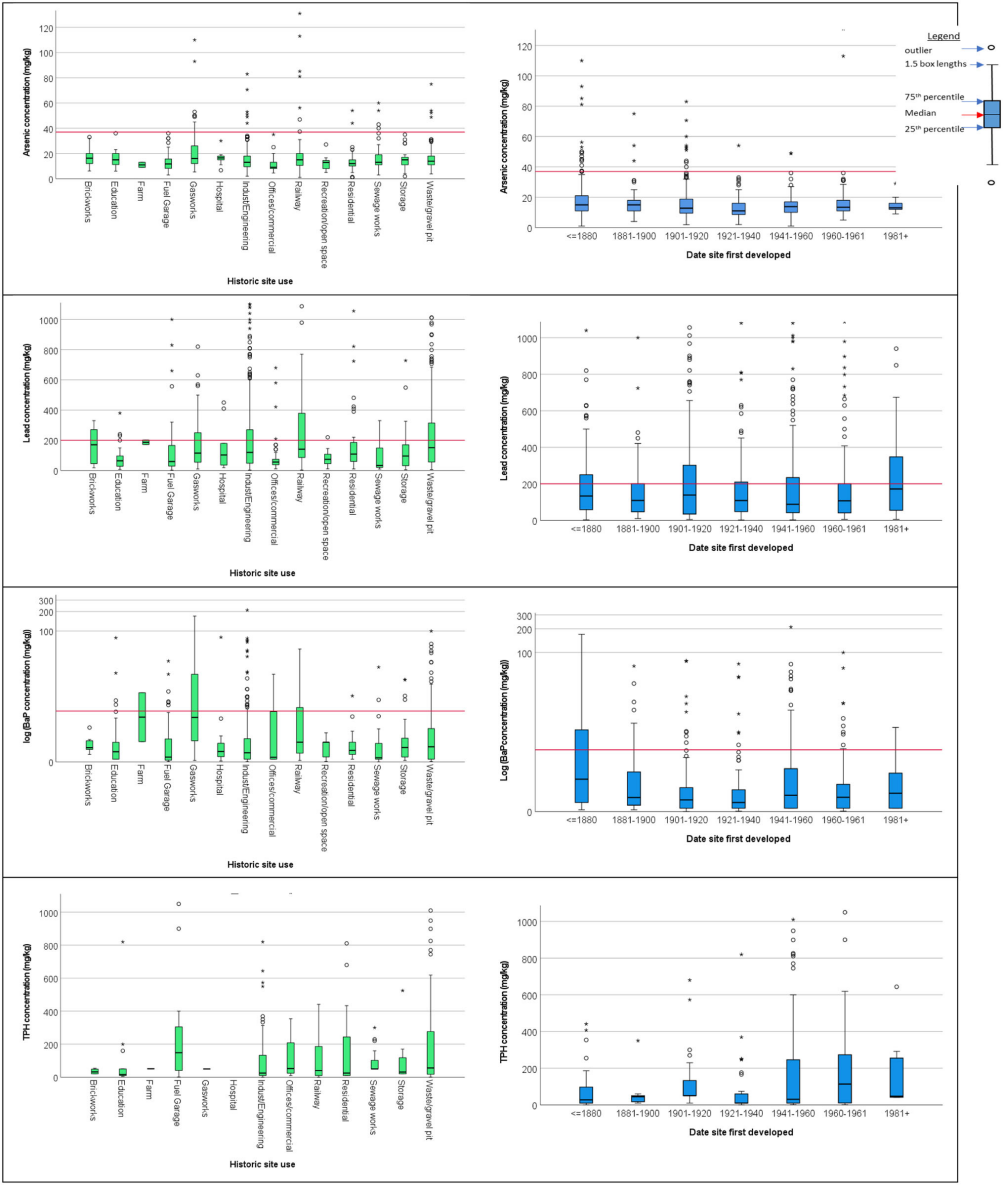
PTE/POP concentrations with historic site use and age site was developed.  indicates UK guidance level for residential use with plant uptake. (Guidance is not available for TPH)

Understanding the link between former site use and age and PTE/POP concentrations will help developers and regulators predict the contamination risk of a redevelopment site. The results for past site use and initial site development on measured PTE/organic pollutant values are shown in Fig. 2. (Results for additional PTE are included in Supplementary Information S3.) These box plots clearly indicate that for some contaminants, e.g. BaP, there is a link between certain past uses, notably gasworks, and the concentrations of chemical measured. For other PTE, e.g. arsenic, the links are less defined, as ranges and medians for most site uses are relatively similar. The main difference between past site use for arsenic is in the number of high value outliers found on former industrial sites such as railway and gasworks. Lead measurements were relatively high for most former brownfield uses with the highest median values found on farms, brickworks, railways, waste/gravel pits and industrial sites. Former uses where elevated lead may not have been predicted, but were found included residential and storage facilities, e.g. domestic garages. The organic pollutants, BaP and TPH, did not follow similar trends; BaP was elevated for gasworks and farm sites, whilst TPH was highest on former fuel garage sites.

**Fig. 3:**
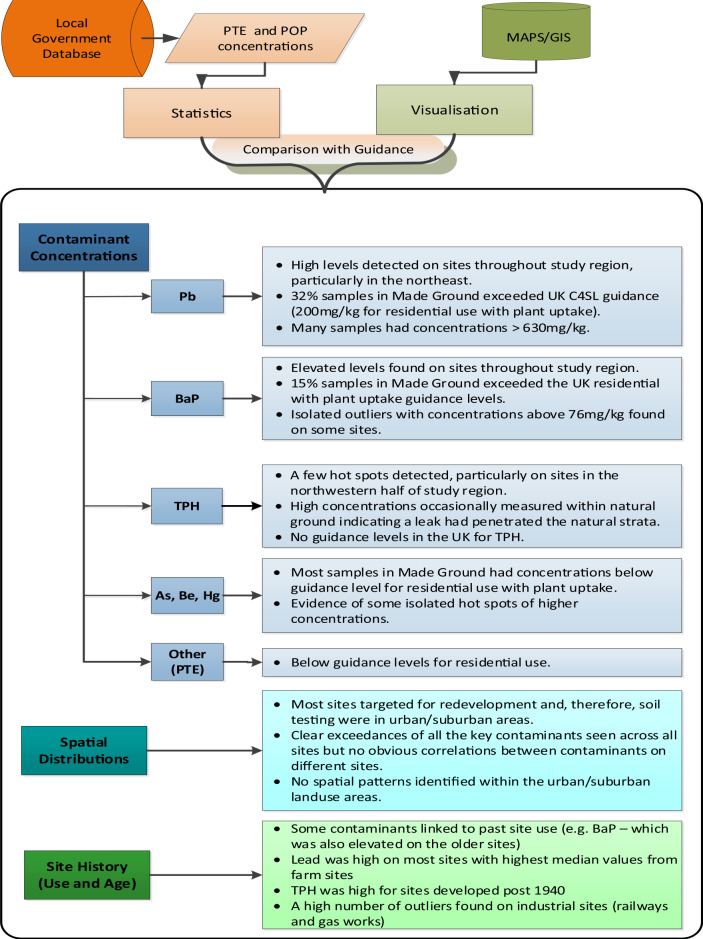
Overall summary of key results for Elmbridge

The median and interquartile results for the relationship between arsenic and the date the site was first developed showed little variation over the time period, however the outlier measurements seem to have reduced considerably between 1920 and 2000. Lead levels seem consistently high throughout the measurement time period, with a high number of outliers and an increase for the 1981+ measurement. (These data were affected by a former farm development where fly tipping of waste was suspected.) Due to the range of values measured, the BaP result is shown on a logarithmic scale. For this plot, the high values prior to 1880, were predominantly for land adjacent to railways that may have been exposed to considerable ash deposition over this extensive time period. In contrast, the TPH showed the highest concentrations for sites developed post 1940. The combined findings from the statistical analysis, visual representation and site history (use and age) for Elmbridge are summarised in Fig. 3.

Lead, BaP and TPH were all detected at elevated levels across the region. There were a number of exceedances for arsenic, beryllium and mercury in Made Ground, but for the latter 2 elements, it should also be noted that the guidance values in Made Ground are relatively low. For the remaining PTE/POP, elevated levels were not detected. There was no evidence that the exceedances of UK guidance levels followed a spatial pattern. There were some links identified with historic site use, in particular for BaPs and for the number of outliers present. Site age was more relevant in older sites with industrial usage linked to fossil fuel combustion, e.g. railways/gasworks (and even some residential sites) where ash (associated with high BaPs) would have been deposited.

## Potential Applications of the Local Government Planning Contamination Dataset

The results presented provide an overview of PTE/organic pollutant concentrations found on brownfield sites in a local government area in Surrey, UK. Resources and time pressures currently prevent collation and interrogation of these data in local government, though advances could be made if the submitted information was digitised. This study demonstrates how this information can be utilised to increase understanding of brownfield contamination and its relationship to historic site use and age. The initial results in terms of site historic use, PTE/POP location and site age alone do not produce relationships that can be used to directly predict PTE/POP behaviour; hence in this section the relevance of this information and how it could be used is explored further.

Local government officers do not have the time or resources to develop complex statistical analysis of this information. It is therefore important to evaluate what is the key information that can be extracted and best practice for display and understanding of local contamination data. Simple box plots provide the key PTE information and can be quickly produced. These, supplemented by GIS maps, can provide a good overview of local contamination data. An example is provided below to highlight these applications for the case study area.

**Fig. 4:**
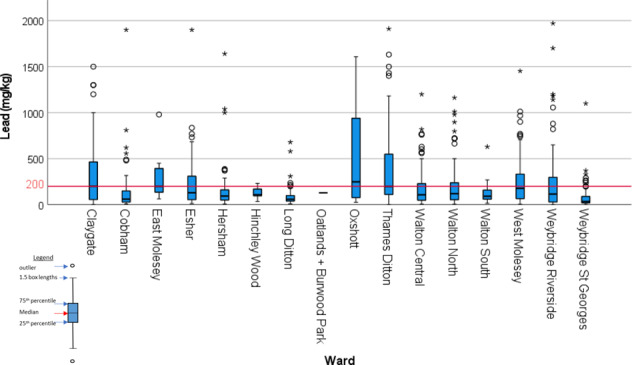
Box plots of lead data in Made Ground for Elmbridge Wards

In the planning system, local government areas are subdivided into electoral wards and thus the contamination data can be evaluated for each ward. Figure 4 provides an overview of lead concentrations obtained on sites within each of the wards in Elmbridge. The median and range of lead values are clearly displayed using a box plot figure. It shows that the levels of lead are elevated throughout Elmbridge Borough, with the median above the current UK guidance for residential use in 2 wards, Claygate and Oxshott.

**Fig. 5:**
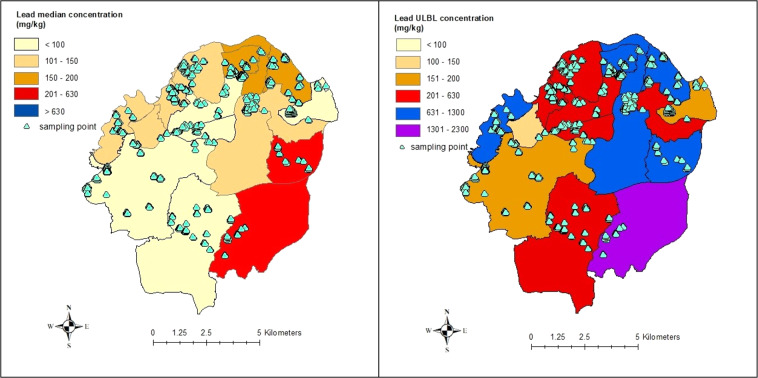
Median and ULBL values of lead in Elmbridge electoral wards and sampling locations. Key: Exceeds UK guidance for:  Residential use with plant uptake,  Public open space (residential),  Public open space (park). (Service Layer Credits: Contains UK data ©Crown Copyright and Database Right 2021)

These results can also be shown spatially (using maps/GIS) as shown in Fig. 5. These could indicate where exceedances have been measured and if there are geographical (or through map comparison, geological) influences on the results. In the example above, it can be quickly ascertained that the highest lead values were found on sites in a ward in the southeast of the region. However, care must be taken when interpreting this ward mapping technique as it is limited in scope. In the case study shown above, the location of sampling points was added to indicate that (i) the number of sites in each ward varied considerably and (ii) the site investigation data were not randomly (or uniformly) scattered throughout each ward but instead were highly clustered and sparsely distributed within a ward. Consequently, neither the lack of uniformity in the spatial distribution nor the density of sampling points have been captured or presented in these maps. Therefore, in this case, the key value for this type of visualisation would be limited to alerting the council to the level of brownfield contamination found in each ward. The individual sampling point plots shown in Figs. 1 and 4 are thus vastly superior. In regions where a systematic, uniform grid sampling approach has been adopted, this ward mapping visualisation method would be appropriate and would capture the correct spatial patterns thus providing a clear visual representation across a region (for example for UK, G-Base Survey, BGS 2022).

Results such as these provide useful “snapshots” of contamination levels at a given time and location. They do not provide any predictive capabilities regarding contaminant concentrations or how they may be distributed spatially or dispersed temporally based on site history or on the date when the site was first developed. The data can, however, be used to evaluate individual site use enrichment factors in which the levels of contamination measured are compared with the background levels. These can then be used to indicate values of potential concentrations of PTE/POP contaminants in the region as described below.

UK legislation (DEFRA 2012) defines the background concentration as a reflection of typical and widespread levels and includes low-level diffuse pollution and common human activity. This part of the 2012 legislation has not been practically implemented in local government, partly due to limited resources and a lack of clear guidance of an appropriate method. Ander et al. (2013) initially measured key contaminants across the UK and developed a method for evaluating the background level. Although rigorous, the method required expert assessment and application (Reimann and de Caritat 2017). Other methods including median +2MAD (median absolute deviation) and the Upper Limit GeoChemical Baseline (ULBL) are more straightforward and have been found to be applicable to urban data (Rothwell and Cooke, 2015; McIlwaine et al. 2017). The ULBL has been used extensively as an indication of the geochemical background (Jarva et al. 2010, McIlwaine et al. 2017). It is evaluated from P_75_ + 1.5(P_75_ −P_25_) where P_75_ and P_25_ are the 75th and 25th percentiles of the element concentrations, respectively (Jarva et al. 2010; Tukey 1977).

Both the median +2MAD and ULBL methods for evaluating the background concentration were applied to the natural soil results for the key contaminants to determine a geogenic background concentration for this dataset. To evaluate a ‘normal’ background concentration as defined in the UK legislation, the ULBL and median +2MAD methods were applied to Made Ground samples for sites considered to have a past use with ‘typical’ human activity. Sites with a former use of education, offices, residential, recreation and storage were considered applicable to this criterion. The results are shown in Table 3. In addition, the enrichment factor, or ratio between ‘normal’ background and geogenic background was evaluated for the key PTE/POPs.

**Table 3:** Background concentrations for the study area for natural soil and ‘normal’ site use

PTE/POP	Geogenic background concentration (natural soil)		‘Normal’ soil concentration		‘Normal’ enrichment factor: $\frac{{{\mathrm {normal}}\,{\mathrm {background}}\,{\mathrm {concentration}}}}{{{\mathrm {Geogenic}}\,{\mathrm {background}}\,{\mathrm {concentration}}}}$	
	Median + 2MAD (mg/kg)	ULBL (mg/kg)	Median + 2MAD (mg/kg)	ULBL (mg/kg)	Median + 2MAD (mg/kg)	ULBL (mg/kg)
Arsenic	20.6	29.5	20.7	27.8	1.0	0.9
Chromium	37	53.5	29	39.5	0.8	0.7
Copper	15.6	25	35	58	2.2	2.3
Lead	30	59	161	287	5.4	4.9
Mercury	0.7	2.3	0.8	1.4	1.1	0.6
Nickel	26	44	23	33	0.9	0.8
Zinc	59	94	164	251	2.8	2.7
BaP	0.2	0.19	1.3	2.2	6.5	11.7
PaH	3.2	4.7	13	23	4.1	4.9
TPH	86	110	52	128	0.6	1.2

There is considerable difference between the background calculation results for Made Ground and geogenic (natural soil) using the two methods. The Median + 2MAD method removed the highest number of outliers and thus produced the most conservative (lowest) value in terms of an upper limit for normal background concentration. These results are similar to those of Rothwell and Cooke (2015) for the urban area of Gateshead in which the data were also taken from a local government dataset. McIlwaine et al. (2017) suggested the Median + 2MAD method may be too conservative and the ULBL is more appropriate. The enrichment factors for copper, lead, zinc, BaP, PaH and TPH exceed 1 and show that these PTE/POP are significantly affected by diffuse pollution and the ‘normal’ human activities on these sites. The particular PTE/POPs affected may indicate that ‘normal’ human activity on these sites includes the spreading of ash from open fires, reflecting the UK’s former dependency on coal based domestic fuel. For the other PTE, where the factor is close to unity, the concentration of PTE found in Made Ground was essentially that of the underlying natural soil.

The geogenic background concentrations represent the results from the natural soil samples in the case study dataset. There were six different natural soil formations identified in this area, which would have different chemical constituents. However, to interpret overall trends, these results were combined to evaluate regional geogenic (or natural soil) background concentrations. The individual geogenic values for the natural soils were also calculated and are shown in Supplementary Information S4.

The background methodology was then applied to the remaining (non ‘normal’) site uses from the earlier study to evaluate individual site use enrichment factors for the key PTE/POPs and the results for the ULBL method are shown in Table 4. The site use enrichment factor is the ratio of (site use background ULBL)/ (normal background ULBL). The results show the PTE/POPs that are predominantly affected by the individual sites uses. For example, gasworks has a major impact on BaP and PAH concentrations, railway use affects copper, BaP, PAH and TPH, and former sewage sites show high levels of chromium. (A similar table for the Median +2MAD method is shown in Supplementary Information S5).

**Table 4:**
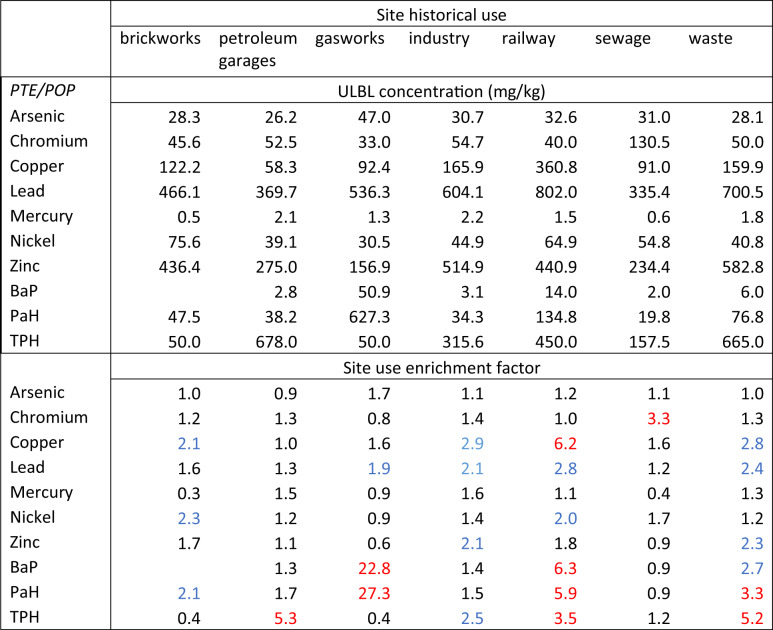
Background concentrations and enrichment factors for key PTE/POP and site historical use

Impact level;  = elevated,  = significant

The results of this investigation can be combined to provide guidance to local developers and regulators of the values of expected concentrations of these PTE/POP on brownfield sites in this region. A suggested results format for the case study of Elmbridge Borough that could be made available to local government (e.g. contaminated land/planning officers) is presented in Table 5.

**Table 5:**
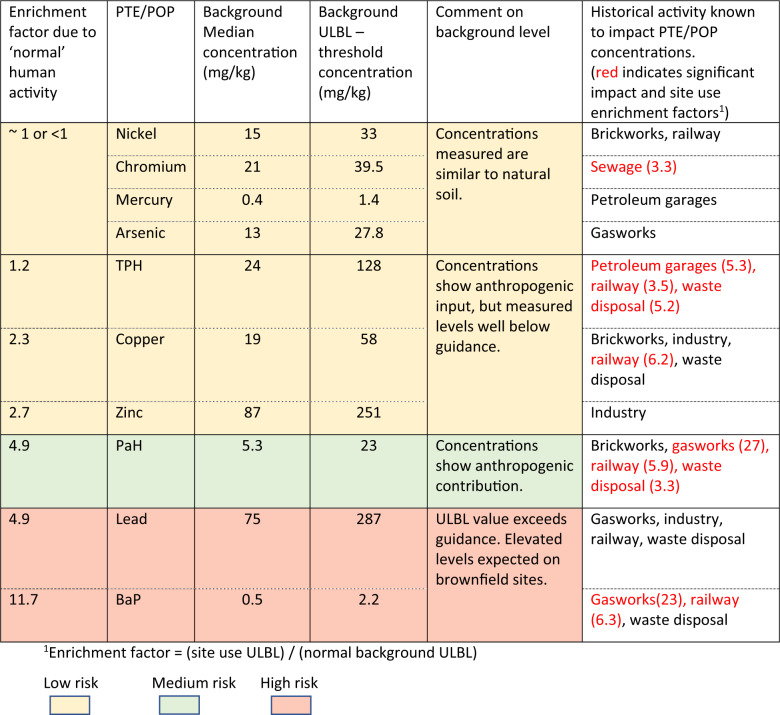
Summary of results and preliminary risk assessment for brownfield sites within Elmbridge

**Fig. 6:**
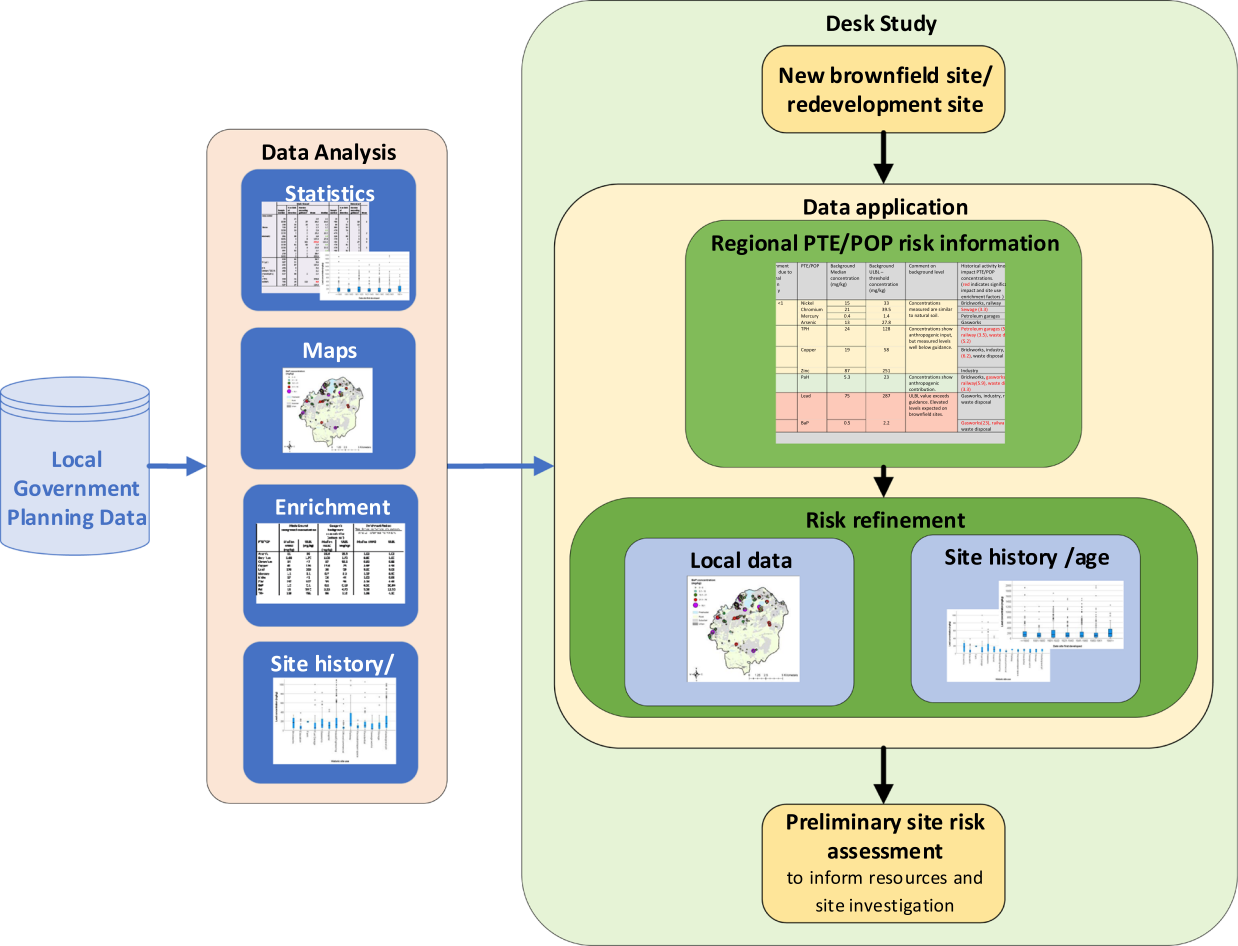
Schematic of the desk study process to determine a preliminary risk assessment for a new brownfield redevelopment site

This table could also be used by developers as part of the desk study process to make a preliminary risk assessment for a new brownfield redevelopment site. As this is generated by the local government, there may be potential liability issues that would need to be considered both by the generator of the table (local government) and the end user. This could be addressed by caveats clearly specifying the authenticity and/or the reliability of the information received to collate this table. Figure 6 shows a schematic of this process which would utilise the analysis of the local government planning data sets. Once analysed, a summary of the local government results (Table 5) could be made available to any redevelopment application. These results can be consulted to provide a regional assessment of PTE/POP levels, which can then be refined further based upon local data visualisation and the box plot/ statistical information for the site history and site age. This would provide a comprehensive picture of the scale and spatial location of contaminants that have already been identified in the region.

The key benefit to local government from the collation of data and summary table (as shown in Table 5), would be that the contaminated land officer would have direct, instant access to information on the potential PTE/POP levels on new re-development sites submitted to them through planning. This would, in turn, inform their contaminated land conditions, expedite their work and identify/highlight the PTE/POPs that should be included by consultants in desk studies, risk assessment and site investigations. For this area, the data has clearly indicated that elevated lead and BaP are likely on brownfield sites. Conversely, very little arsenic contamination was found. This information would also inform the local authority’s UK Part 2A legislation requirements for prioritising sites for further investigation (DEFRA 2012). In addition, these results provide information to planners and policy makers on potential site uses for local development plans.

Contaminated land can be a sensitive issue with the public and can affect house sales (Syms 1998). Consequently, there could be a negative public reaction to greater information on regional PTE/POP levels, particularly for more familiar PTEs, e.g. arsenic. This may impact local responses to new developments, influence planning applications, increase development time and lead to economic impacts, such as fewer residential sales. However, to place this work in context, the information acquired from this study is directed towards identifying the potential for higher risk PTE/POP that might be found on a site prior to investigation and remediation. It is, therefore not targeted towards public dissemination but instead directed as additional assistance for contaminated land officers to ensure the safe remediation of their brownfield development sites. It should be noted that a significant proportion of residential sites in the UK are built on brownfield sites, hence previous contamination is likely and contamination issues rarely attract media attention (Ministry of Housing, Communities and Local Government 2019).

For the developer, this local area summary provides preliminary information at the initial stage in a site development. It can therefore identify potential PTE/POP risks and inform potential development costs enabling appropriate resources and contingency plans to be included within project management. Unexpected contamination found later in a project can incur significant costs and delays (WHO 2021). This summary should be the starting point for a desk study and preliminary risk assessment leading to more targeted site investigation design. A further benefit is the information can inform health and safety requirements and highlight onsite risks for the site investigation team. The negative aspect of providing more generalised regional data is that developers and consultants may consider this reduces the need for intrusive investigation. The results have confirmed the range of PTE/POPs found and the lack of uniformity across site ages, uses and regions and thus reinforce the on-going need for intrusive investigation on brownfield sites in the UK.

## Conclusions

This interrogation of the local government dataset has provided detailed information to inform regulators, developers and consultants on potential PTE/POP concentrations on brownfield sites in the study area. The dataset was therefore shown to be a valuable resource. Exploitation of this resource would be enhanced and expedited with digitisation of data at the planning submission stage.

Box plots indicating statistical results, together with GIS maps of upper bound PTE/POP measured levels are suggested as optimum ways of visualising the results. Enrichment factors provided clear indicators of where PTE/POP concentrations have been influenced by anthropogenic factors. The results confirm that due to the range of PTE/POPs found and the lack of predictive capability from existing data, that it is essential to continue with intrusive site investigations on any new brownfield redevelopment sites in the UK.

The analysis and results were brought together in a final summary table for the PTE/POP in the study area and a desk study process schematic to incorporate this information in the assessment of new sites. The authors believe the summary table and desk study application have benefits for developers/regulators assessing brownfield sites. This is by the early identification of potential PTE/POP risks enabling the appropriate allocation of resources, development of contingency plans and evaluation of health and safety requirements. These benefits can be fully evaluated through direct consultation with practitioners, both in local government and the commercial sector.

## Supplementary Information

Supplementary Information

## Data Availability

Due to UK data protection law the information from this research is not available from the authors.
