# Supplementary material for: Evaluation and Potential Uses of Site Investigation Soil Contamination Data Submitted to Uk Local Government
Source: Environ Manage. 2022 May 4;70(1):1–15. doi: 10.1007/s00267-022-01648-1 (PMC9160098; doi:10.1007/s00267-022-01648-1)
Supplement: Supplementary file 1 — Supplementary Information [file 267_2022_1648_MOESM1_ESM.docx]

SUPPLEMENTARY MATERIAL

S1: Details on proposed site use and current site land use classification for Elmbridge dataset.

S2: Comparison of statistical results from all samples or per site.

| Chemical | Made Ground | | | | | | | Natural soil | | | | | | |
| --- | --- | --- | --- | --- | --- | --- | --- | --- | --- | --- | --- | --- | --- | --- |
|  | Number of samples | % at limit of detection | Number exceeding guidance | Mean per sample | Median per sample | Mean per site | Median per site | Number of samples | % at limit of detection | Number exceeding guidance | Mean per sample | Median per sample | Mean per site | Median per site |
| *Units mg/kg unless stated* |  |  |  |  |  |  |  |  |  |  |  |  |  |  |
| Antimony | 46 | 24 |  | 2.8 | 2.0 | 2.7 | 2.7 | 16 | 56 |  | 1.3 | *1.0* | 1.3 | *1.3* |
| Arsenic | 1008 | 2 | 37 | 16.0 | 13.0 | 16.4 | 13.8 | 481 | 2 | 13 | 13.5 | 11.0 | 14.8 | 12.2 |
| Beryllium | 238 | 35 | 15 | 1.1 | 1.0 | 1.0 | 1.0 | 98 | 50 | 12 | 1.4 | *1.0* | 0.9 | *0.8* |
| Water soluble Boron | 715 | 37 | 0 | 1.7 | 1.0 | 1.5 | 1.0 | 355 | 53 | 0 | 1.1 | *0.8* | 1.3 | *1.0* |
| Cadmium | 1008 | 53 | 0 | 0.9 | *0.5* | 0.8 | *0.5* | 479 | 76 | 0 | 0.6 | *0.5* | 0.6 | *0.5* |
| Chromium | 1008 | 0 | 0 | 28.2 | 22.0 | 26.6 | 21.9 | 479 | 0 | 0 | 28.1 | 23.0 | 26.6 | 23.0 |
| Chromium (hexavalent) | 351 | 98 | 0 | 1.9 | *1.0* | 2.4 | *2.0* | 116 | 98 | 0 | 2.0 | *2.0* | 2.3 | *2.0* |
| Copper | 1005 | 3 | 8 | 137.3 | 27.0 | 106.4 | 31.5 | 479 | 8 | 1 | 30.5 | 9.2 | 32.3 | 12.6 |
| Lead | 1008 | 0 | 321 | 255.0 | 110.0 | 229.5 | 139.6 | 481 | 4 | 27 | 97.8 | 18.0 | 123.8 | 35.0 |
| Mercury | 1008 | 61 | 62 | 0.7 | *0.5* | 0.7 | *0.5* | 479 | 81 | 0 | 0.5 | *0.3* | 0.5 | *0.4* |
| Nickel | 1008 | 2 | 3 | 21.8 | 17.0 | 21.9 | 17.4 | 479 | 4 | 1 | 18.8 | 15.0 | 18.3 | 17.0 |
| Selenium | 984 | 82 | 0 | 1.4 | *1.0* | 1.5 | *1.0* | 465 | 82 | 0 | 1.3 | *1.0* | 1.5 | *1.0* |
| Vanadium | 279 | 0 | 0 | 38.4 | 36.0 | 34.9 | 37.0 | 116 | 0 | 0 | 39.8 | 37.4 | 35.7 | 36.0 |
| Zinc | 1000 | 0 | 3 | 200.5 | 94.0 | 182.8 | 115.0 | 477 | 1 | 0 | 82.3 | 41.0 | 102.8 | 51.2 |
| Total Cyanide | 808 | 82 |  | 36.7 | *1.0* | 6.9 | *1.0* | 352 | 93 |  | 2.2 | *1.0* | 2.4 | *1.0* |
| Sulphate as SO4 ( g/l) | 587 | 14 |  | 9.6 | 0.1 | 4.8 | 0.1 | 232 | 20 |  | 2.0 | 0.0 | 1.3 | 0.0 |
| Sulphide | 430 | 67 |  | 120.4 | *10.0* | 139.2 | *8.0* | 239 | 89 |  | 25.2 | *10.0* | 21.8 | *8.0* |
| Organic Matter % | 245 | 3 |  | 3.1 | 2.3 | 3.2 | 2.5 | 91 | 9 |  | 1.8 | 1.4 | 1.7 | 1.5 |
| Total Organic Carbon (TOC) % | 365 | 4 |  | 2.0 | 1.2 | 2.0 | 1.5 | 124 | 23 |  | 0.8 | 0.4 | 1.0 | 0.7 |
| Total Phenols (monohydric) | 807 | 86 | 0 | 1.4 | *1.0* | 1.1 | *1.0* | 357 | 96 | 0 | 0.9 | *1.0* | 1.0 | *1.0* |
| Total Petroleum Hydrocarbons (TPH) | 349 | 41 |  | 376.9 | 45.0 | 281.5 | 71.6 | 208 | 63 |  | 244.5 | 31.9 | 301.2 | 46.0 |
| Benzo(a)pyrene (BaP) | 755 | 26 | 112 | 8.8 | 0.5 | 4.4 | 0.9 | 346 | 68 | 6 | 1.7 | 0.1 | 1.2 | 0.1 |
| Total PAH | 827 | 27 |  | 114.3 | 6.8 | 52.8 | 10.7 | 379 | 68 |  | 115.7 | 1.6 | 92.2 | 2.0 |

^a^Guidance is either C4SL or S4UL for residential use with plant uptake (DEFRA 2014, Nathanail et al. 2015)

**Bold** values show where a value exceeds the current UK guidance for residential use with plant uptake.

*Italic* indicates where median is also the limit of detection for the PTE

| 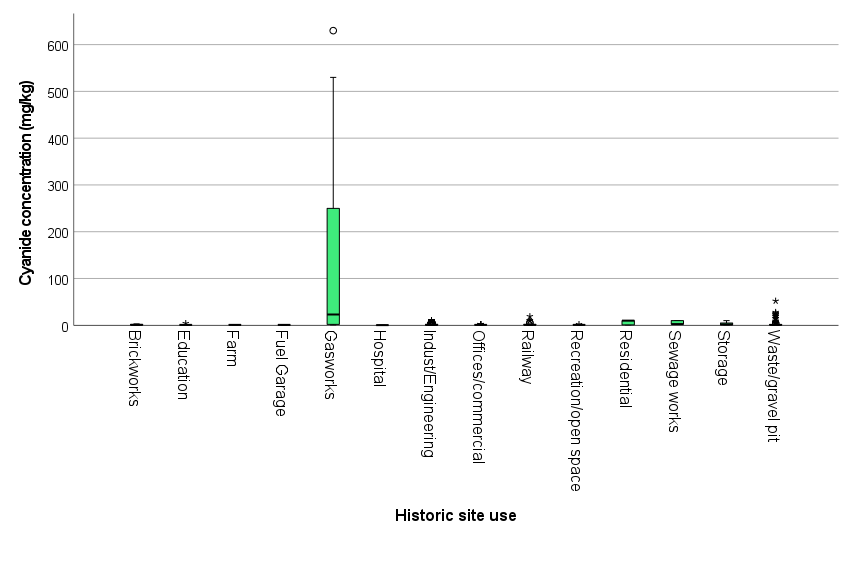 | 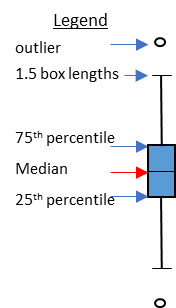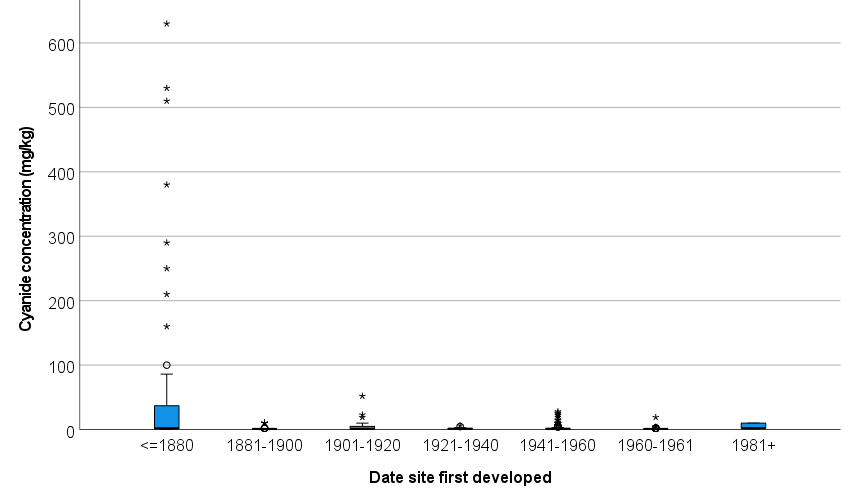 |
| --- | --- |
| 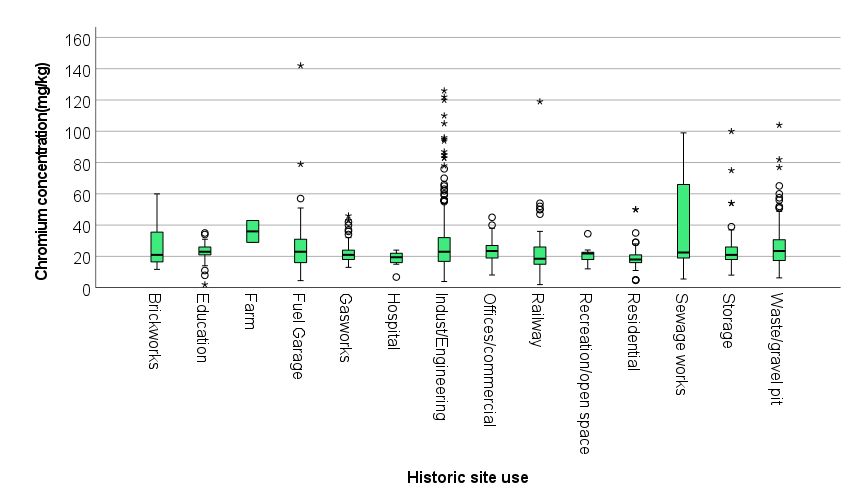 | 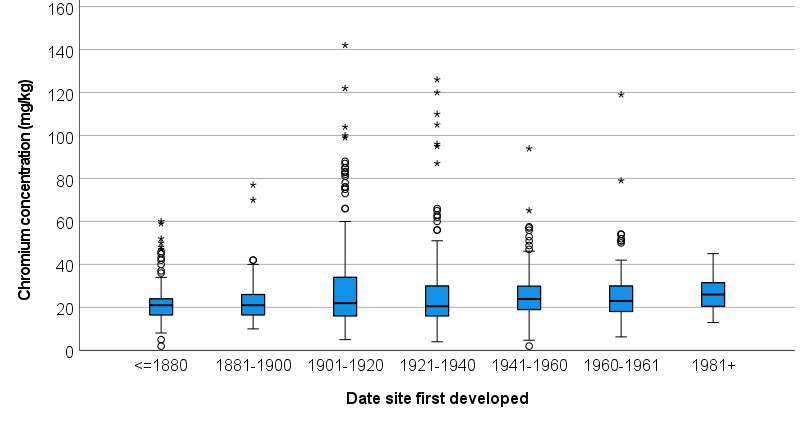 |
| 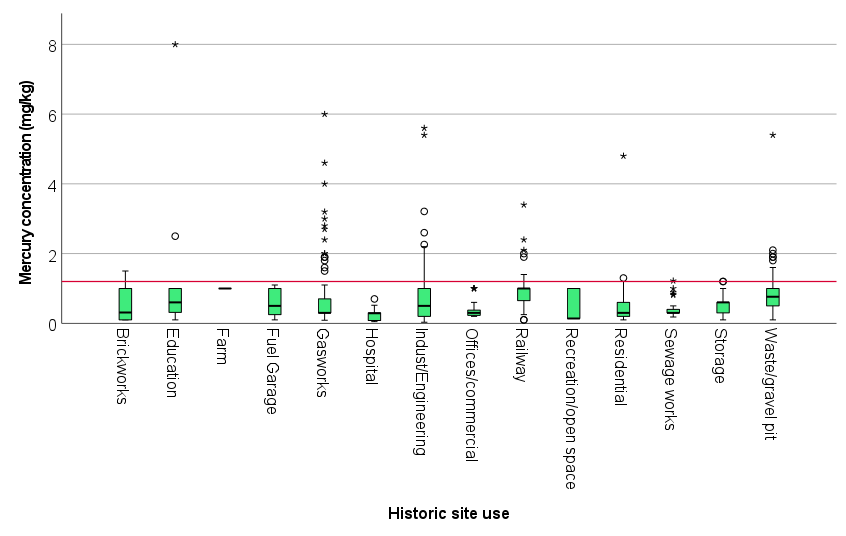 | 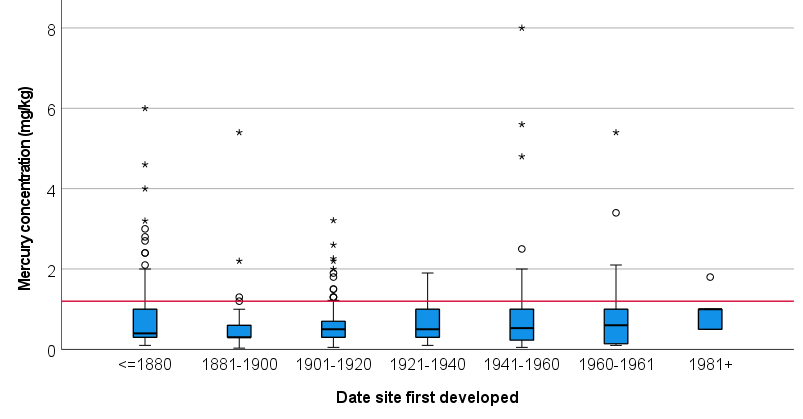 |

S3 PTE concentrations with historic site use and age site was developed.

indicates guidance level for residential use with plant uptake.

(For chromium VI guidance level = 910mg/kg; guidance not available for cyanide,)

S4 Background (ULBL) concentrations of PTE in natural underlying soils

|  | Alluvium | Bagshot | Kempton Park Gravel | Taplow  Gravel | Claygate | London Clay | All soils |
| --- | --- | --- | --- | --- | --- | --- | --- |
| Arsenic | 31.5 | 30.6 | 26.5 | 32.6 | 20.4 | 25.3 | 29.5 |
| Chromium | 43.0 | 57.1 | 51.1 | 53.8 | 27.9 | 49.5 | 53 |
| Copper | 27.5 | 18.7 | 22.2 | 23.6 | 9.3 | 26.3 | 25 |
| Lead | 59.7 | 32.9 | 65.6 | 59.5 | 42.3 | 48.0 | 58.7 |
| Mercury | 2.4 | 0.6 | 2.2 | 0.8 | 1.4 | 2.1 | 2.3 |
| Nickel | 40.6 | 31.9 | 44.1 | 47.5 | 10.0 | 53.0 | 43.8 |
| Zinc | 112.3 | 68.6 | 103.5 | 74.0 | 57.6 | 108.2 | 94 |
| BaP | 0.2 | 0.1 | 0.1 | 0.2 | 0.2 | 2.4 | 0.19 |
| PAH | 3.8 | 4.0 | 3.8 | 4.8 | 22.9 | 3.7 | 4.8 |
| TPH | 516.3 | 110.0 | 90.0 | 84.5 | - | 35.6 | 110 |

S5 Table 4. Background concentrations and enrichment factors for key PTE/POP and site historical use. Impact level; blue = elevated, red = significant

|  | Site historical use | | | | | | |
| --- | --- | --- | --- | --- | --- | --- | --- |
|  | brickworks | petroleum garages | gasworks | industry | railway | sewage | waste |
| *PTE/POP* | Median +2MAD concentration (mg/Kg) | | | | | | |
| Arsenic | 23.4 | 19.1 | 26.0 | 21.0 | 24.0 | 21.0 | 20.3 |
| Chromium | 29.1 | 37.0 | 27.0 | 38.8 | 27.5 | 52.7 | 36.5 |
| Copper | 87.5 | 35.0 | 41.0 | 85.0 | 82.5 | 26.0 | 76.6 |
| Lead | 138.0 | 147.1 | 277.0 | 294.0 | 309.0 | 64.5 | 369.5 |
| Mercury | 0.1 | 1.1 | 0.3 | 1.2 | 1.4 | 0.3 | 1.2 |
| Nickel | 28.1 | 17.5 | 24.0 | 30.0 | 30.0 | 37.0 | 28.8 |
| Zinc | 92.1 | 142.2 | 109.5 | 236.0 | 211.5 | 142.5 | 282.9 |
| BaP |  | 0.5 | 11.2 | 1.0 | 2.8 | 0.3 | 1.9 |
| PaH | 30.0 | 10.5 | 133.8 | 14.0 | 28.3 | 4.9 | 21.5 |
| TPH | 20.0 | 387.1 | 50.0 | 72.4 | 116.0 | 50.0 | 149.3 |
|  | Enrichment factor | | | | | | |
| Arsenic | 1.1 | 0.9 | 1.3 | 1.0 | 1.2 | 1.0 | 1.0 |
| Chromium | 1.0 | 1.3 | 0.9 | 1.3 | 0.9 | 1.8 | 1.3 |
| Copper | 2.5 | 1.0 | 1.2 | 2.4 | 2.4 | 0.7 | 2.2 |
| Lead | 0.9 | 0.9 | 1.7 | 1.8 | 1.9 | 0.4 | 2.3 |
| Mercury | 0.1 | 1.4 | 0.4 | 1.5 | 1.8 | 0.4 | 1.5 |
| Nickel | 1.2 | 0.8 | 1.0 | 1.3 | 1.3 | 1.6 | 1.3 |
| Zinc | 0.6 | 0.9 | 0.7 | 1.4 | 1.3 | 0.9 | 1.7 |
| BaP |  | 0.4 | 8.6 | 0.7 | 2.2 | 0.2 | 1.5 |
| PaH | 2.3 | 0.8 | 10.3 | 1.1 | 2.2 | 0.4 | 1.7 |
| TPH | 0.4 | 7.4 | 1.0 | 1.4 | 2.2 | 1.0 | 2.9 |
